# Resilience of bumblebee foraging behavior despite colony size reduction

**DOI:** 10.3389/finsc.2022.1073380

**Published:** 2023-01-04

**Authors:** Maxence Gérard, Justine Marchand, Jade Zanutto, Emily Baird

**Affiliations:** ^1^ INSECT Lab, Division of Functional Morphology, Department of Zoology, Stockholm University, Stockholm, Sweden; ^2^ Sorbonne Université, Faculté des Sciences et Ingénierie, Paris, France

**Keywords:** *Bombus terrestris*, eusociality, pollination, social group size, visiting rate

## Abstract

Foraging behavior is driven by diverse factors, notably life history traits. Foraging strategies are particularly complex among eusocial species such as bumblebees, because they depend primarily on the needs of the colony, rather than on individual’s needs. Colony size, i.e. the number of workers in a colony vary a lot among eusocial insects. While a large colony can be adaptive, several drivers can strongly decrease colony size, like pesticides or high temperatures. In this study, we used the bumblebee *Bombus terrestris* to assess if workers adapted their foraging behavior to such rapid decreases in colony size. We conducted the foraging experiments with two plant species commonly used by bumblebees: *Borago officinalis* and *Echium plantagineum*. Several foraging parameters were measured: foraging time, number of foraging trips, number of workers foraging, handling time and visiting rate. Despite a drastic reduction in colony size, nearly all the foraging behavior parameters were unaffected by the colony size reduction. Colonies that were subject to a large decrease in workers instead displayed high resilience and behavioral plasticity by quickly increasing the proportion of foragers. Ultimately, further research should assess if this consistency in foraging behavior also allows bumblebee colonies to maintain both the efficiency of the resources collection and pollination.

## 1 Introduction

Understanding foraging behavior, and the decisions made by animals when collecting food requires consideration of the energy spent foraging and the energy gained by the nutrient intake (1, 2). However, foraging behavior strategies can be influenced by diverse factors such as life history traits (e.g. parental care, degree of sociality, etc.; 3, 4). Indeed, foraging behavior is even more complex in social animals, where not only the individual fitness has to be considered, but also the fitness of the group (Galef & 5, 6). In eusocial species, such as honeybees and bumblebees, the foraging behavior of the nonreproductive workers is defined primarily by the needs of the colony, rather than by the foraging individual’s physiological needs (7).

Among eusocial bees, colony size can range from some tens of workers for the smallest bumblebee colonies up to several tens of thousands for honeybees (8). In bumblebees, a large colony size is often adaptive. By increasing their colony size, bumblebees can increase their foraging range (9) and thus the amount of resources they can gather (10). Larger colonies can also increase their reproductive success by producing more sexual brood (11, 12) and they are more resilient to stressors (13, 14). Smaller colonies would be more vulnerable to individual errors, as they have fewer additional workers to recruit if individual mistakes during the foraging are made (*e.g.* a worker failing to efficiently gather resources and return with them to the colony), or if food intake is too low. However, bumblebee colony size is constrained by several factors. Firstly, habitat appears to set strong limits on colony size – for example, some arctic species can have colonies with only 20 workers (15–17), while tropical species can have colonies with more than one thousand workers (15, 16). Secondly, colony size varies temporally, starting with extremely few workers after the colony is first founded by the queen and reaching a peak population by the summer (9). Colony size is also dependent of the availability of resources (9, 10) and parasitism (18). In addition, anthropogenic drivers can lead to selection episodes and colony mortality. Bumblebee colonies are particularly sensitive during the early stages of their life cycle, and stressors such as pesticides (19) and high developmental temperatures (20) can impede colony growth, leading to smaller colonies or to a drastic reduction of an existing colony.

Colonies can adapt to reductions in the number of workers by, for example, extending their growth phase (21). However, the extent to which workers will adapt their foraging behavior to a reduction colony size is mostly unknown. Among the few studies assessing changes in foraging behavior due to colony reduction, Beekman et al. (22) showed that, in honeybees, foraging distance decreased with worker number but that the number of floral patches visited (proportionally to the number of workers in the colony) increased. However, it remains unclear if the proportion of workers recruited to forage was significantly higher in smaller colonies or if, instead, the workers from these smaller colonies were performing significantly more trips per individual. Using the bumblebee *Bombus terrestris*, Biella et al. (23) highlighted that, despite a reduction of the workforce, the diversity of plants collected remained roughly the same and that the foraging activity of the workers in reduced colonies significantly increased. These few studies could indicate that the foraging behavior of social bees is resilient to drastic reductions in colony size, but the effect of this reduction on a wider range of foraging parameters remains to be investigated.

To better understand how variations in colony size affect foraging behavior, we investigate how reduced colony size influences several foraging parameters in the buff-tailed bumblebee *Bombus terrestris*. Previous studies have indicated that reductions in worker number can occur as a result of exposure to a stressor (e.g. heat; 20, 24) and that this, in turn, lead to changes in foraging behavior. However, whether these changes were produced by physiological effects of the stressor *per se*, by colony size reduction, or the combination of these two variables is unknown. We tested the hypothesis that changes in colony size alone (i.e. without any additional stressor) affect bumblebee foraging behavior. To do this, we manipulated the colony size of *B. terrestris* such that they had a normal number of workers (100) and colonies that had half this number (50). We then placed the colonies in a large flight room and allowed the workers to freely forage on two different types of plants, *Borago officinalis* and *Echium plantagineum*. Foraging behavior was analyzed by recording the following parameters: (i) foraging time, (ii) number of foraging trips, (iii) handling time, (iv) visiting rate, (v) number of workers foraging. In agreement with a study performed in field conditions (23), we hypothesize that either the proportion of workers foraging from the reduced colonies, or the number of foraging trips per individual could increase in response to the reduction of the work force. Indeed, Pendrel & Plowright (25) showed that colony size reduction could lead to the increase of the larval feeding rate, and that the behavioral plasticity of bumblebee workers could allow a reallocation of tasks to recruit more foragers (26, 27).

## 2 Materials and methods

### 2.1 Model species

We purchased *Bombus terrestris* colonies that were housed in plastic boxes (28 cm x 25 cm x 20 cm) from the company Koppert (Berkel en Rodenrijs, The Netherlands). Both bumblebee rearing and plant-pollinator experiments were conducted at the Tovetorp Zoological Research Station of Stockholm University (Sweden). Until the start of the foraging experiments, the colonies were maintained at 27°C, an optimal developmental temperature for bumblebees (28, 29) and 50% humidity and had access to a sucrose solution (Koppert Natupol Smart) provided by the commercial breeder. The bumblebees in this experiment had spent the entirety of their lives in the colony and had therefore no foraging experience prior to these experiments. Every three to five days, colonies were also fed with an ad libitum quantity of commercial fresh-frozen organic pollen (Naturprodukter, Raspowder Bipollen), finely crushed, and mixed with a 50% sucrose solution.

### 2.2 Colony reduction

A total of eight hives were used in the experiments, which were conducted between April and June 2022. As it was not possible to test all hives simultaneously, we performed two identical sessions using four colonies per session (i.e. the maximal number of colonies that the flight room could accommodate at a time).

To test the effects of colony size on foraging behavior, the colonies were subjected to one of two different treatments: normal colonies (our control condition), where the number of workers was reduced to 100 (an amount commonly encountered in wild colonies) and small colonies, where the worker population was decreased to 50, representing a significant reduction of the population size but one that would still include enough workers to measure foraging behavior. All colonies contained the queen and all existing cells and brood. By reducing the workers in the normal size treatment, we could also control for the effect of individual’s manipulation during the colony reduction and the marking. All the colonies had between 114 and 135 workers before being reduced. The average colony reduction was 18.03% for the normal colonies, and 60.32% for the small colonies. For each experimental session, we included two colonies from the small size treatment and two colonies from the normal size treatment. Each worker was marked individually by gluing a number plate to their thorax.

### 2.3 Foraging experiment

Two plant species were selected for the foraging experiments: *Borago officinalis* and *Echium plantagineum*. We chose these plant species because of their attractiveness, their widespread distribution throughout Europe, and because their separated flowers allow to accurately measure the foraging parameters (30–33). For both plant species, the seeds were provided by Impecta Fröhandel AB (Julita, Sweden). They were placed in a germination room under a constant temperature of 19°C and a 16h light:8h dark photoperiod. As soon as seedlings had three leaves, they were transplanted into 2 L pots filled with a 1:2 (v/v) mix of sand (size 0.8-1.2 mm, Ardex, Witten, Germany) and universal peat compost (Plantagen, Kongsvinger, Norway). The plants were then grown in a controlled room at a constant temperature of 24°C, a relative humidity of 80% and watered daily. In total, 20 plants of each species were monitored. Plant growth lasted 10 weeks.

We conducted the foraging experiments in a 6 m x 6 m x 3 m flight room, maintained at a constant temperature, luminosity and relative humidity (24°C, 2050 lx and 25%, respectively), under a 16:8 h light/dark cycle. Two colonies from each size treatment (4 colonies in total) were placed 60 cm apart on a 60 cm high table at 4 m from the plant pots. The bees could enter the colony *via* a landing platform and a plastic tube 5 cm long and 1 cm in diameter. Microscope cameras connected to a computer record every entrance and exit of the bees. To allow the bees to get accustomed to the room and the flowers, the colonies were placed in the flight room and opened (so that the bees could start to forage freely) 1 day before each experimental session. We measured the foraging parameters during the following four days for each plant species – in session 1, *E. plantagineum* was presented for the first four days and *B. officinalis* was presented on the next four days, in session 2 this order was reversed.

Foraging behavior was assessed using several parameters. Foraging time, the number of foraging trips (per individual and per treatment) and the number of workers foraging were acquired over 7 h each day from the recordings of the microscope camera using the software OBS (34). Foraging time was measured as time in minutes, between the exit and the return of a worker. We only selected the foraging time of workers returning to their own colony (some workers would occasionally enter a colony that was not their own). The number of foraging trips per individual was calculated as the number of times an individual worker departed and returned to its own colony per day (*i.e*. one value per individual per day). The total number of foraging trips per colony size treatment was also calculated for the whole experiment, as well as the total number of workers foraging. Handling time and visiting rate were measured by following the workers with a chronometer from 9 am to 4 pm during each of the 16 experimental days. Handling time was defined as the time spent from the first contact with a flower to the last contact with this same flower. Visiting rate was defined as the number of flowers visited by a worker within 1 min. For these two parameters, we removed the extreme points (i.e. defined as values above upper quartile 3 + 3 x interquartile range or below lower quartile Q1 – 3x interquartile range), because they did not correspond to biologically relevant measurements (e.g. a worker staying on a flower for a long time without gathering any food resources).

### 2.4 Statistical analyses

We assessed the impact of colony size on the different foraging parameters using generalized linear mixed models (GLMM) and the lmer4 R package (35). We ran separate models for each foraging parameter and for both plant species (to account for any differences between the flower types). For each analysis, we started with the full model including the foraging parameters as response variable, the colony size treatment as a fixed effect and colony ID (the individual identifier of each colony from 1 to 8), individual ID (nested in colony ID, allowing us to account for pseudoreplication), as well as session number as random factors. The final model was selected using the lowest AICc across all possible model combinations (which always included colony size treatment). If the ΔAICc < 2, we used the simplest model. As the distribution of the residuals was not normal (even after testing different type of data transformation) for the foraging time and the handling time for both plant species, as well as for the visiting rate using *E. plantagineum*, we used a Gamma distribution, which was the most accurate distribution for non-normal continuous data. Finally, for the number of foraging trips, we used a Poisson distribution, which is appropriate for count data.

## 3 Results

### 3.1 Foraging behavior – foraging time

**Figure 1:**
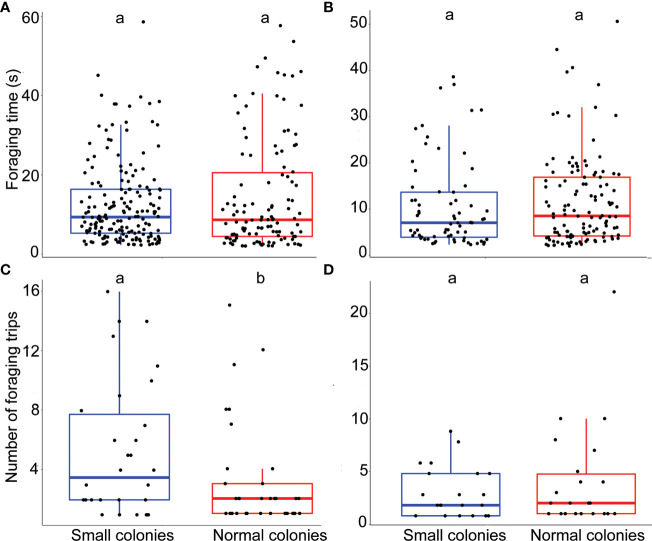
Impact of colony size on bumblebee foraging behavior. **(A)** Foraging time (s) for *E. plantagineum* (n = 301). **(B)** Foraging time for *B. officinalis* (n = 169). **(C)** Number of foraging trips for *E. plantagineum* (n = 63). **(D)** Number of foraging trips for *B. officinalis* (n = 43). The data for the number of foraging trips correspond to the number of trips made by each individual per day. Letters at the top of the boxplots indicate significant differences when the letters are different.

The model that best explained the variation in foraging time of *Echium plantagineum* (n = 291) included colony size treatment as a fixed factor and individual ID as a random factor (next best model ΔAICc 2, Supplementary material, **Table S2-3** for details of the model). We did not observe any significant impact of colony size treatment on foraging time (p = 0.13; **Figure 1A**). The random factor individual ID (nested in colony ID) explained 0.2% of the variance that remained in the residuals.

The model that best explained the variation in foraging time of *Borago officinalis* (n = 169) included colony size treatment as a fixed factor (next best model ΔAICc 1.11, Supplementary material, **Table S1** for details of the model). We did not observe any significant impact of colony size treatment on foraging time (p = 0.52; **Figure 1B**).

### 3.2 Foraging behavior – number of foraging trips

**Figure 2:**
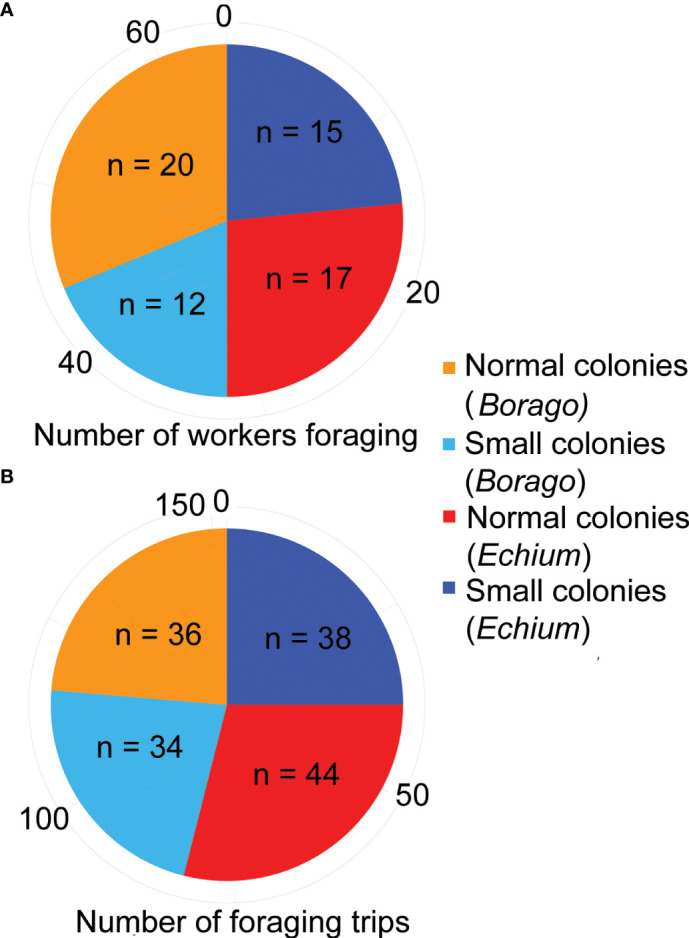
**(A)** Total number of workers performing foraging trips per treatment and per plant species. **(B)** Total number of foraging trips per treatment and per plant species. Only workers that foraged on flowers and returned to their own colony were included.

We first counted the total number of foraging trips per colony size treatment (**Figure 2A**). In total, 40 workers from the smaller colonies performed 72 trips, while 52 workers from the normal size colonies performed 80 trips (**Figures 2A, B**). Thus, 47% of the recorded foraging trips were performed by workers from the smaller colonies. Out of a total of 200 workers in the small size colonies, 20% of them were observed to efficiently perform foraging trips and visit flowers, while this value was only 13% for the normal size colonies.

The model that best explained the variation in the number of foraging trips of *E. plantagineum* (n = 63) included colony size treatment as a fixed factor and individual ID nested in colony ID as a random factor (next best model ΔAICc 0.81, Supplementary material, **Table S5** for details of the model). The number of foraging trips was significantly higher in the smaller colonies (p < 0.001; r-squared = 0.72; **Figure 1C**).

The model that best explained the variation in the number of foraging trips of *B. officinalis* (n = 43) included colony size treatment as a fixed factor and individual ID and session as random factors (next best model ΔAICc 0.2, Supplementary material, **Table S4** for details of the model). The number of foraging trips was not significantly affected by the colony size treatment (p = 0.6; r-squared = 0.7; **Figure 1D**).

### 3.3 Foraging behavior – handling time

**Figure 3:**
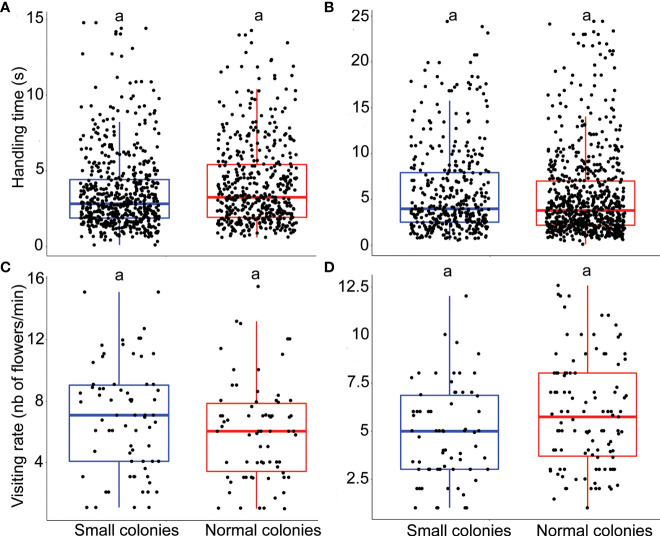
Impact of colony size on bumblebee foraging behavior. **(A)** Handling time (s) for *E. plantagineum* (n = 1068). **(B)** Handling time (s) for *B.officinalis* (n = 1135). **(C)** Visiting rate for *E. plantagineum* (n = 140). **(D)** Visiting rate for *B. officinalis* (n = 174). Letters at the top of the boxplots indicate significant differences when the letters are different.

The model that best explained the variation in handling time of *E. plantagineum* (n = 1068) included colony size treatment as a fixed factor and individual ID as a random factor (next best model ΔAICc 0.1, Supplementary material, **Tables S8-9** for details of the model). Handling time for *E. plantagineum* was not significantly affected by colony size (p = 0.36; **Figure 3A**). The random factor individual ID explained 0.8% of the variance that remained in the residuals.

The model that best explained the variation in handling time of *B. officinalis* (n = 1135) included colony size treatment as a fixed factor and individual ID nested in colony ID as a random factor (next best model ΔAICc 2, Supplementary material, **Tables S6-7** for details of the model). There was no significant effect of colony size on handling time for *B. officinalis* (p = 0.907; **Figure 3B**). The random factor individual ID (nested in colony ID) explained 0.31% of the variance that remained in the residuals.

### 3.4 Foraging behavior - visiting rate

The model that best explained the variation in visiting rate of *E. plantagineum* (n = 140) included colony size treatment as a fixed factor and individual ID as a random factor (next best model ΔAICc 1.65, Supplementary material, **Tables S12-13** for details of the model). Visiting rate for *E. plantagineum* was not significantly affected by colony size (p = 0.77; **Figure 3C**). The random factor individual ID explained 1.1% of the variance that remained in the residuals.

The model that best explained the variation in visiting rate of *B. officinalis* (n = 174) included colony size treatment as a fixed factor and individual ID as a random factor (next best model ΔAICc 2, Supplementary material, **Tables S10-11** for details of the model). Visiting rate for *B. officinalis* was not significantly affected by colony size (p = 0.223; **Figure 3D**). The random factor individual ID explained 0.77% of the variance that remained in the residuals.

## 4 Discussion

The goal of this study was to assess if a drastic reduction in colony size would affect the foraging behavior of bumblebees. Surprisingly, our analyses showed that, with the exception of the number of foraging trips on *E. plantagineum*, none of the aspects of foraging behavior we measured were affected by colony size. This is not consistent with previous studies, which have showed that stressors strongly affected several aspects of foraging behavior. For example, exposure to elevated temperature during development caused an increase in worker visiting rate and the number of foraging trips, while the handling time and the foraging time decreased, suggesting that workers were trying to maximize the collection of resources in stressful conditions (20). Interestingly, in this previous work, the number of workers produced in the colonies that were exposed to higher developmental temperatures was reduced, so it was not possible to assess if the changes in foraging behavior were the consequence of a direct effect of temperature on the physiology/behavior of workers, or if the main driver of these changes was the decrease in colony size. The results of this present study suggest that the reduction in colony size alone was not enough to explain changes in foraging behavior observed among bumblebees that developed at an elevated temperature.

When focusing solely on colony size reduction, several studies have indicated that foraging behavior is robust to changes in worker number. Interestingly, in field conditions, Biella et al. (23) highlighted that a removal of 50% of bumblebee workers in a colony lead to an increase in the number of foraging trips of the remaining workers. This increase of activity resulted in the reduced colonies having a similar diet to the non-reduced colonies. Together with our findings, this study suggests that, independently of the experimental conditions (field conditions *versus* controlled conditions), reduced colonies can compensate for the loss of workers by increasing the foraging activity of their workers. Among honeybees, smaller colonies of *Apis mellifera* forage on approximately the same number of flower patches as larger colonies by reducing the number of workers visiting each patch (rather than increasing the proportion of foragers; 22). We observed a different pattern in our study: the proportion of workers foraging from the smaller colonies was higher than in the larger colonies. Indeed, while around 20% of the workers from the small colonies were observed visiting the flowers, only 13% of the workers from the larger colonies were visiting them (**Figure 2A**). Thus, while the number of trips per worker in the smaller colonies was higher in only one of the two plant species, the proportion number of workers from smaller colonies recruited to forage was higher overall. The role assigned to workers inside a bumblebee colony is not as fixed as it is in honeybee colonies, and previous studies have shown that bumblebee workers can switch between tasks (25, 36). For example, depending on the resource requirements, some workers can switch from nursing to foraging, although specialized foragers are more efficient at their task and specialized nurses are faster at feeding the brood (26, 37). Even within foragers, Hagbery and Nieh (38) showed that, when some pollen specialist foragers were removed from the colony, generalist foragers could focus on pollen foraging to compensate this lost. We can thus assume that some workers that were not specialized in foraging initially in the smaller colonies became foragers to compensate for the reduced colony size.

Foraging bumblebee workers are also constrained by the quantity of resources brought back in the colony and the quantity of nectar stored in the honey pots – the motivation to forage increases when the quantity of nectar in these pots decreases (39, 40). Interestingly, two studies on honeybees showed that removing pollen stores could increase the proportion of workers foraging in the colony (41, 42). This could explain why we observed a higher proportion of foraging bumblebees in the small colonies in our study: as we did not manipulate the number of cells or larvae, there were still many larvae to feed but a lower worker force to bring back resources, which could have reduced the proportion of pollen stored and lead to the recruitment of more foragers. Indeed, it is known that the size of the brood has an impact on the quantity of pollen brought back to the colony (43). It would thus be interesting to assess if changes in both the size of the brood and the number of workers would ultimately affect the foraging behavior. Another interesting point to assess would be to compare reduced colonies with unmodified colonies. In our framework, we decided to control for the colony size in every colony from the “normal size” treatment, to have a similar number of workers among these colonies (*i.e.* 100 workers) and logistical limitations prevented us from testing the three treatments simultaneously (*i.e.* drastically reduced, slightly reduced and unmodified). However, an interesting next step would be to repeat this experiment with half of the colonies unmodified. Based on the results of Biella et al. (23) who did not modify the number of workers in the “normal size” colonies, we could hypothesize that the colonies reduced to 50 workers would compensate for the loss in workforce and have similar foraging parameters to the unmodified colonies.

How colony size impacts the foraging behavior of eusocial insects probably depends on the information used by foragers to localize, reach their food resources and recruit other foragers. For example, Pharaoh’s ants lay pheromone trails to bring back their food sources to the nest. In this species, smaller colonies had a more disorganized foraging system: foragers from these colonies failed to reach a feeder as often as those from larger colonies (44). This impact is probably due to the volatility of the pheromone trails: if too few ants use the trail, it is not maintained and individuals will forage more erratically. In that case, colony size thus had a crucial impact on the foraging behavior and performance. In bumblebees, individual workers must rely primarily on their own ability to locate and learn about profitable floral resources (45). While the efficiency of ant foraging strategy is strongly relying on chemical cues, bumblebee ability to rely primarily on individual learning seem to confer them a higher resilience in their foraging behavior.

As our results indicate that bumblebee foraging behavior is not affected by colony size, the next step would be to assess if the quantity of resources collected and the efficiency of its collection also remain similar. In the desert seed-harvester ants, larger colonies were not more efficient at gathering food from clumped resources, and their foraging rate also remained constant despite variations in colony size (46). This indicates that eusocial insects are quite robust to colony size reduction and are not only able to keep their foraging behavior constant but also their efficiency at collecting resources. However, this assumption remains to be tested in bumblebees, and is particularly important to assess considering that they will be subjected to more stressors caused by human activities in the future. Indeed, while bumblebee foraging behavior was resilient to colony size reduction, other individual or colony features may have been affected by this reduction. For example, Müller and Schmid-Hempel (47), showed that bumblebee colony reduction could affect female body size and decrease the number of males produced. This could ultimately affect the fitness of colonies in the next generation, a hiding cost that should be considered in future experiments.

## Data availability statement

The original contributions presented in the study are included in the article/**Supplementary Material**. Further inquiries can be directed to the corresponding author.

## Author contributions

MG and EB conceived the ideas. MG, JM, JZ and EB contributed to the design of the methodology. JM and JZ collected the data. MG, JM and JZ analyzed the data. MG led the writing of the manuscript with critical input from EB. All authors contributed to the article and approved the submitted version.
