# Supplementary material for: Resilience of bumblebee foraging behavior despite colony size reduction
Source: Front Insect Sci. 2023 Jan 4;2:1073380. doi: 10.3389/finsc.2022.1073380 (PMC10926374; doi:10.3389/finsc.2022.1073380)
Supplement: Supplementary file 1 [file DataSheet_1.pdf]

Supplementary Material

1 Supplementary Figures and Tables

1.1 Supplementary Tables

Table S1. Generalized linear model output of the best model (lowest AICc) to predict foraging time on *Borago officinalis*.

	Foraging time			
Predictors	Estimates	Std Error	t value	p
(Intercept)	0.086	0.007	12.787	< 0.001
Colony size	0.008	0.012	0.701	0.484
Model structure is Foraging time ~ Colony size				

Table S2. Generalized linear mixed model output of the best model (lowest AICc) to predict foraging time on *Echium plantagineum*.

	Foraging time			
Predictors	Estimates	Std Error	t-value	p
(Intercept)	0.081	0.011	7.528	< 0.001
Colony size	0.021	0.014	1.533	0.125
Model structure is Foraging time ~ Colony size + (1 | ID)				

Table S3. Proportion of variance explained by the random factor in the best linear mixed model predicting foraging time on *Echium plantagineum*.

	Random effects	
Groups	Variance	Std. Deviation
Individual ID	0.0012	0.035
Residual	0.6152	0.784
# Observations	291	
# Individual ID	42	

Table S4. Generalized linear mixed model output of the best model (lowest AICc) to predict the number of foraging trips on *Borago officinalis*.

	Number of Foraging trips			
Predictors	Estimates	Std Error	z-value	p
(Intercept)	1.24	0.311	3.983	< 0.001
Colony size	-0.197	0.371	-0.531	0.596
R-squared fixed effect		0.01		
R-squared random effect		0.69		
Model structure is NbForagingTrips ~ Colony size + (1 | ID) + (1 | session)				

Table S5. Generalized linear mixed model output of the best model (lowest AICc) to predict the number of foraging trips on *Echium plantagineum*.

	Number of Foraging trips			
Predictors	Estimates	Std Error	z-value	p
(Intercept)	0.902	0.002	374.6	< 0.001
Colony size	0.567	0.002	235.5	< 0.001
R-squared fixed effect		0.05		
R-squared random effect		0.66		
Model structure is NbForagingTrips ~ Colony size + (1 | Colony/ID)				

Table S6. Generalized linear mixed model output of the best model (lowest AICc) to predict Handling Time on *Borago officinalis*.

	Handling Time			
Predictors	Estimates	Std Error	t-value	p
(Intercept)	0.178	0.02	8.794	<0.001
Colony size	-0.004	0.031	-0.117	0.907
Model structure is HandlingTime ~ Colony size + (1 | Colony/ID)				

Table S7. Proportion of variance explained by the random factor in the best linear mixed model predicting Handling Time on *Borago officinalis*.

	Random effects	
Groups	Variance	Std. Deviation
ID:Colony	0.002	0.06
Residual	0.6344	0.796
# Observations	1132	
# ID:Colony	33	

Table S8. Generalized linear mixed model output of the best model (lowest AICc) to predict Handling Time on *Echium plantagineum*.

	Handling Time			
Predictors	Estimates	Std Error	t-value	p
(Intercept)	0.224	0.022	10.336	<0.001
Colony size	0.03	0.029	0.905	0.365

Model structure is HandlingTime ~ Colony size + (1 | ID)

Table S9. Proportion of variance explained by the random factor in the best linear mixed model predicting Handling Time on *Echium plantagineum*.

	Random effects	
Groups	Variance	Std. Deviation
Individual ID	0.004	0.063
Residual	0.488	0.699
# Observations	1066	
# Individual ID	32	

Table S10. Linear mixed model output of the best model (lowest AICc) to predict visiting rate on *Borago officinalis*.

	Visiting rate			
Predictors	Estimates	Std Error	df	p
(Intercept)	0.199	0.022	9.043	< 0.001
Colony size	0.038	0.031	1.218	0.223

Model structure is VisitingRate ~ Colony size + (1 | ID)

Table S11. Proportion of variance explained by the random factor in the best linear mixed model predicting visiting rate on *Borago officinalis*.

	Random effects	
Groups	Variance	Std. Deviation
Individual ID	0.0018	0.042
Residual	0.23	0.48
# Observations	174	
# Individual ID	30	

Table S12. Linear mixed model output of the best model (lowest AICc) to predict visiting rate on *Echium plantagineum*.

	Visiting rate			
Predictors	Estimates	Std Error	t-value	p
(Intercept)	0.199	0.024	8.247	<0.001
Colony size	0.01	0.035	0.292	0.77

Model structure is VisitingRate ~ Colony size + (1 | ID)

Table S13. Proportion of variance explained by the random factor in the best linear mixed model predicting visiting rate on *Echium plantagineum*.

	Random effects	
Groups	Variance	Std. Deviation
Individual ID	0.003	0.051
Residual	0.235	0.484
# Observations	140	
# Individual ID	33	
